# Genetics versus environment in the pathophysiology of sagittal synostosis

**DOI:** 10.1007/s00381-026-07396-5

**Published:** 2026-07-18

**Authors:** Valentina De Gregorio, Domiziano D. Tosi, Alessia Vita, Martina Salvati, Luca Massimi, Gianpiero Tamburrini, Federica Tiberio, Wanda Lattanzi

**Affiliations:** 1 Dipartimento Di Scienze Della Vita E Sanità Pubblica, Università Cattolica del Sacro Cuore, Rome, Italy; 2 Unità Operativa Complessa Di Neurochirurgia Infantile, Fondazione Policlinico Universitario A. Gemelli IRCSS, Rome, Italy; 3 Dipartimento Di Neuroscienze, Università Cattolica del Sacro Cuore, Rome, Italy; 4 Fondazione Policlinico Universitario A. Gemelli IRCSS, Rome, Italy

**Keywords:** Sagittal craniosynostosis, Scaphocephaly, Non-syndromic craniosynostosis, Genetics, Geneticist, Environmental risk factors, Prenatal exposure

## Abstract

**Purpose:**

This systematic review aims to provide an updated overview of the relative contribution of germline genetic and environmental factors to the pathophysiology of sagittal craniosynostosis (sCS).

**Methods:**

Using the PubMed database in accordance with Preferred Reporting Items for Systematic Reviews and Meta-Analyses (PRISMA) guidelines, relevant studies addressing genetic and/or environmental determinants of sCS in paediatric patients were systematically reviewed.

**Results:**

A total of 1238 records were identified, of which 97 studies met inclusion criteria after full-text review. Overall, 25,877 patients were included, with 11,086 diagnosed with sCS (42.8%). Germline genetic variants potentially associated with craniosynostosis were reported in 251 patients, accounting for 12.6% of genetically tested individuals with sCS. Identified variants were distributed across 125 genes, with recurrent findings in 25 genes. Environmental and perinatal factors were more consistently reported across large population-based and case-control studies. Frequently associated factors included male sex, advanced maternal age, maternal smoking, thyroid dysfunction, fertility treatments, foetal constraint, prematurity, and abnormal birth weight, although effect sizes varied across studies.

**Conclusion:**

Identifiable germline genetic causes appear to account for only a minority of sCS cases, whereas epidemiological evidence suggests that environmental and perinatal influences may also contribute to disease pathogenesis. Nevertheless, most cases lack identifiable germline mutations in key signalling pathways, and the available evidence does not support either genetic or environmental factors as individually deterministic drivers of disease development. Overall, the evidence synthesized in this review supports a multifactorial model for sCS, in which rare genetic susceptibility and non-genetic influences likely interact in the etiopathogenesis of the condition rather than reflecting a single dominant aetiology. However, heterogeneity across studies, differences in genetic testing methodologies, potential bias in exposure assessment, and the limited availability of integrative analyses restrict definitive conclusions regarding the relative contribution of these factors.

**Graphical Abstract:**

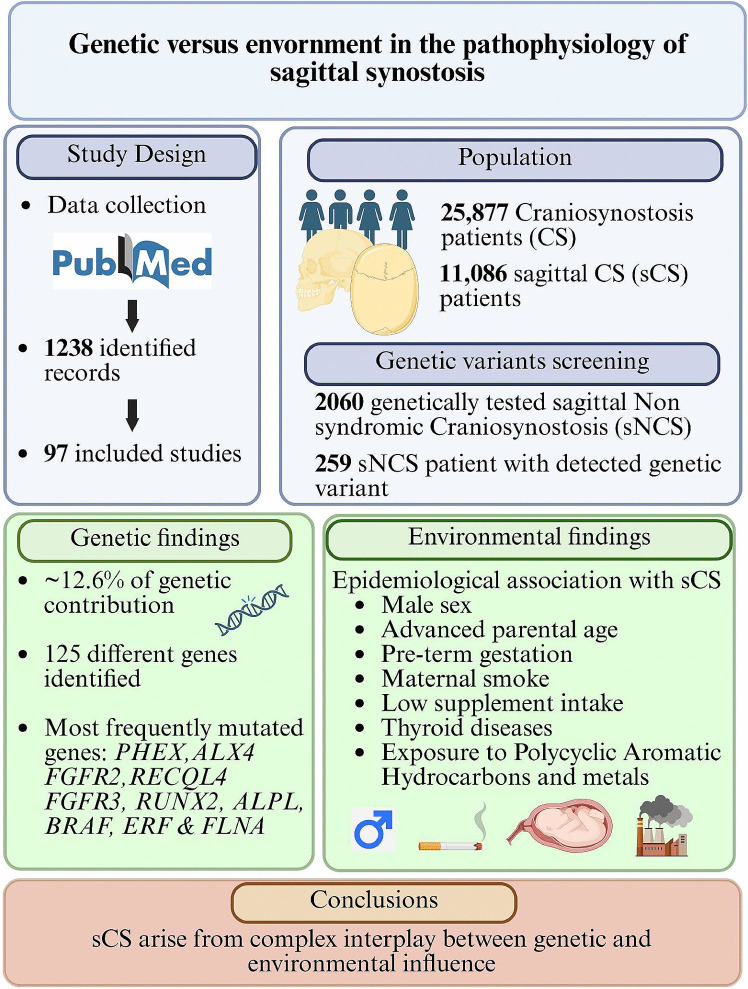

**Supplementary information:**

The online version contains supplementary material available at 10.1007/s00381-026-07396-5.

## Introduction

Craniosynostosis (CS) is a group of rare craniofacial disorders affecting approximately 1:2500 live births [1, 2]. It is a clinically and genetically heterogeneous congenital condition characterized by the premature fusion of one or more cranial sutures.

Depending on the suture involved, CS results in characteristic skull shape abnormalities and is classified as scaphocephaly (sagittal synostosis), trigonocephaly (metopic synostosis), brachycephaly (bicoronal synostosis), or anterior/posterior plagiocephaly (unicoronal or lambdoid synostosis) [3–6]. 

CS may present either as part of a syndrome, with multiple suture involvement and additional extracranial malformations and/or developmental delay, or as an isolated condition. Syndromic forms account for approximately 25–30% of cases and are commonly associated with monogenic mutations in key osteogenic genes including *FGFR1-3* (*Fibroblast Growth Factor Receptor 1–3*), *TWIST1* (*Twist Family BHLH Transcription Factor 1*), *EFNB1*(*Ephrin-B1*), and *ERF* (*ETS2 Repressor Factor*) [7–10]. In contrast, non-syndromic CS (NCS) typically involves a single suture and represents approximately 70–75% of all cases [11, 12]. Both genetic predisposition and environmental influences contribute to NCS aetiology [12, 13].

Among NCS, sagittal CS (sCS) is the most common form, accounting for 40–60% of all CS cases, with an estimated incidence of 1:5000 live births [14]. It occurs more frequently in males and is typically diagnosed in the neonatal period through clinical examination [15, 16]. sCS is characterized by an elongated and narrow skull (scaphocephaly), with frontal and occipital bossing and reduced biparietal width, without significant facial or skull base asymmetry.

Although sagittal synostosis is often considered a relatively homogeneous condition, increasing evidence suggests considerable phenotypic variability [17]. The sagittal suture does not fuse according to an all-or-nothing mechanism; rather, different segments of the suture may be variably involved, resulting in distinct morphological patterns largely explained by compensatory growth mechanisms, as described by Virchow’s law [14, 17–20]. These include dolichocephaly (complete suture fusion), leptocephaly (anterior third involvement), bathrocephaly (anterior and middle thirds), cynocephaly (middle third), and sphenocephaly (middle and posterior thirds) [14, 17–20].

The severity and pattern of deformation are influenced not only by the extent of suture involvement but also by the timing of suture fusion during prenatal development. Early fusion is generally associated with more severe deformities, whereas later fusion results in milder phenotypes [5]. These compensatory mechanisms may persist even after surgical correction and can influence surgical outcomes.

Both genetic predisposition and environmental factors are thought to contribute to the aetiology of non-syndromic sCS [12, 13]. Although more than 60 genes have been implicated in CS overall, including *FGFR1–3*, *TWIST1*,* EFNB1*, and* ERF*, the genetic basis of isolated sagittal synostosis remains less clearly defined [21].

Environmental influences have also been proposed as contributing factors, particularly in non-syndromic cases. These include in utero mechanical constraints, maternal health conditions, medication exposure, and other prenatal risk factors [1, 2, 11, 22–25]. Epidemiological studies have identified several demographic and environmental associations, such as male sex, advanced maternal age, and specific prenatal exposures including maternal smoking, hypoxia-related conditions, sodium valproate, retinoic acid, antidepressants, and assisted reproductive technologies [15, 26–35].

Despite these findings, the relative contribution of genetic and environmental factors in the pathogenesis of sagittal synostosis remains unclear. Current evidence is fragmented and often limited by small sample sizes or the investigation of isolated risk factors [10, 29, 31, 36]. Therefore, a comprehensive evaluation of the available evidence is needed to better understand the interplay between genetic susceptibility and environmental influences in the development of sagittal synostosis.

The objective of this systematic review is to critically evaluate and compare current up to date evidence on the genetic versus environmental determinants in sCS.

## Methods

### Study design

This systematic review included non-randomized studies of interventions (NRSI), specifically cohort studies, case-control studies, population-based studies, case series, and case reports. The inclusion of observational study designs was justified by the nature of the research question: aetiological investigations of genetic and environmental factors in sagittal craniosynostosis cannot be addressed through randomized controlled trials for ethical and practical reasons. No randomized controlled trials were identified during the screening process. AMSTAR 2 appraisal tool has been used to assess systematic review quality [37].

### Protocol

A written protocol specifying the research question, population, exposures of interest (genetic variants and environmental/perinatal factors), inclusion and exclusion criteria, planned search strategy, and data extraction plan was established by the authors prior to the commencement of data collection.

### Information sources and search strategy

The PRISMA [38] principle was used as an assessment tool, using the PubMed database to collect data. The search was conducted around four main focuses: craniosynostosis AND scaphocephaly/non-syndromic/single-suture AND genetics OR environmental, using a combination of Medical Subject Headings (MeSH) and keywords. The search was conducted on 31/12/2025. The full-search strategy, which resulted in 1238 records, is detailed in Table 1.

**Table 1:** Search strategy. The details of each of the three focuses of the PubMed search are listed along with the number of results that each search produced

(craniosynostosis[MeSH Terms] OR craniosynostos* OR craniosynostosis)	AND	(scaphocephaly[MeSH Terms] OR scaphocephaly OR scaphocephalic OR “single suture” OR “single-suture” OR sagittal)	AND	((genetic predisposition[MeSH Terms] OR germline mutations[MeSH Terms] OR “genetic disposition” OR “genetic predisposition” OR “germline predisposition” OR “genetic susceptibility” OR “genetic” OR “genetic basis” OR “genetic testing” OR geneticist*)	OR	(environmental exposure[MeSH Terms] OR “environmental factors” OR environment* OR prenatal exposure[MeSH Terms] OR maternal exposure[MeSH Terms] OR paternal exposure[MeSH Terms] OR “maternal age” OR “paternal age” OR “maternal smoking” OR smoking[MeSH Terms] OR alcohol drinking[MeSH Terms] OR “drug exposure” OR “medication exposure” OR antiepileptic* OR “valproic acid” OR “fertility treatment” OR assisted reproductive techniques[MeSH Terms] OR “birth weight” OR low birth weight[MeSH Terms] OR prematurity[MeSH Terms] OR folic acid[MeSH Terms] OR nutrition[MeSH Terms]))
9955 results		66,554 results		2,249,836 results		3,526,288 results

### Eligibility criteria

Eligibility criteria for the title and abstract screening and full-text screening are available in Tables 2 and 3, respectively.

**Table 2:** Eligibility criteria for title and abstract screening. The five exclusion criteria applied in the title and abstract screening phase are listed in the table

Exclusion criteria for title and abstract screening	
1. Genetics not mentioned 2. Environment not mentioned 3. Sagittal suture not mentioned 4. Suture not mentioned 5. Not English language

**Table 3:** Eligibility criteria for full-text screening. The eight exclusion criteria applied in the full-text screening phase are listed in the table

Exclusion criteria for full-text screening	
1. Limited to patients with non-sagittal craniosynostosis 2. Limited to multiple suture craniosynostosis 3. Limited to syndromic craniosynostosis 4. No information on germline genetics 5. Limited to analysis of common germline variants 6. No study participants with craniosynostosis 7. Non-English 8. Non-original research/review	

### Selection and data collection process

 Study selection was conducted in two stages by two independent reviewers (V. DG and D. D. T.) using Rayyan systematic review software [39]: stage 1, title, and abstract screening: All retrieved citations were independently screened by both reviewers using the prespecified eligibility criteria. Studies clearly not meeting inclusion criteria were excluded. Stage 2, full-text assessment: Full texts of potentially eligible studies were retrieved and assessed for eligibility by both reviewers independently. Reasons for exclusion were documented. Disagreement resolution: Any discrepancies between reviewers at either stage were resolved through discussion, and if consensus was not reached, a third senior reviewer adjudicated (F. T.).

Data extraction tables are available in Appendix 1.

### Data collection

Data extraction was performed independently by two reviewers (V.DG. and D.D.T.) using a standardized form. Disagreements were resolved through discussion and consensus (> 80%). For each included study, the following descriptive information were collected: title of study, authors, journal and year of publication, study type, total number of patients, and the number of patients (or animals, when applicable) diagnosed with sCS.

To evaluate the genetic contribution, all molecular analyses reported in the studies were systematically reviewed and collected, recording the techniques used (e.g. targeted sequencing, gene panels, WES/WGS, cytogenetic arrays) and, for each relevant variant, noting its type, genomic position, and predicted functional impact, when available.

In parallel, the assessment of environmental determinants was conducted using bibliographic search criteria based on MeSH Terms and extended keywords. Specifically, studies reporting prenatal or perinatal exposures related to environmental factors were included, such as environmental exposure, maternal/paternal exposure, maternal or paternal age, smoking habits, alcohol consumption, fertility treatments or assisted reproductive technologies, birth weight, prematurity, maternal nutritional status, metal exposure, and folic acid supplementation. All environmental variables were recorded according to the information provided in the individual studies.

### Risk of bias assessment

The methodological quality of included studies was independently assessed by two reviewers (V.DG. and D.D.T.) using the Newcastle-Ottawa Scale (NOS). The NOS was selected as it is a validated tool specifically designed for the quality appraisal of non-randomized observational studies, including cohort and case-control designs. The scale evaluates quality across three domains: selection (0 to 4 stars), comparability (0 to 2 stars), and outcome or exposure (0 to 3 stars), for a maximum score of 9 stars.

Studies were classified as high quality (7 to 9 stars), moderate quality (4 to 6 stars), or low quality (0 to 3 stars). Case reports and case series were not scored using the NOS, as this instrument is not validated for these study designs; instead, they were narratively assessed and classified as inherently carrying a high risk of bias due to the absence of a control group, small sample sizes, and limited generalizability. Discrepancies between the two reviewers were resolved through discussion and consensus. The results of the quality assessment are presented in Appendix 1.

### Synthesis of results

Given the clinical and methodological heterogeneity across the included studies, a narrative synthesis was performed rather than a quantitative meta-analysis. The findings are presented descriptively, organized by aetiological domain (genetic versus environmental determinants), with effect sizes and odds ratios reported where provided by the original studies.

The sources of heterogeneity were explored in the context of differences in study populations, sequencing methodologies, exposure assessment methods, geographic settings, and diagnostic criteria across the included studies.

## Results

### Study selection

**Fig. 1:**
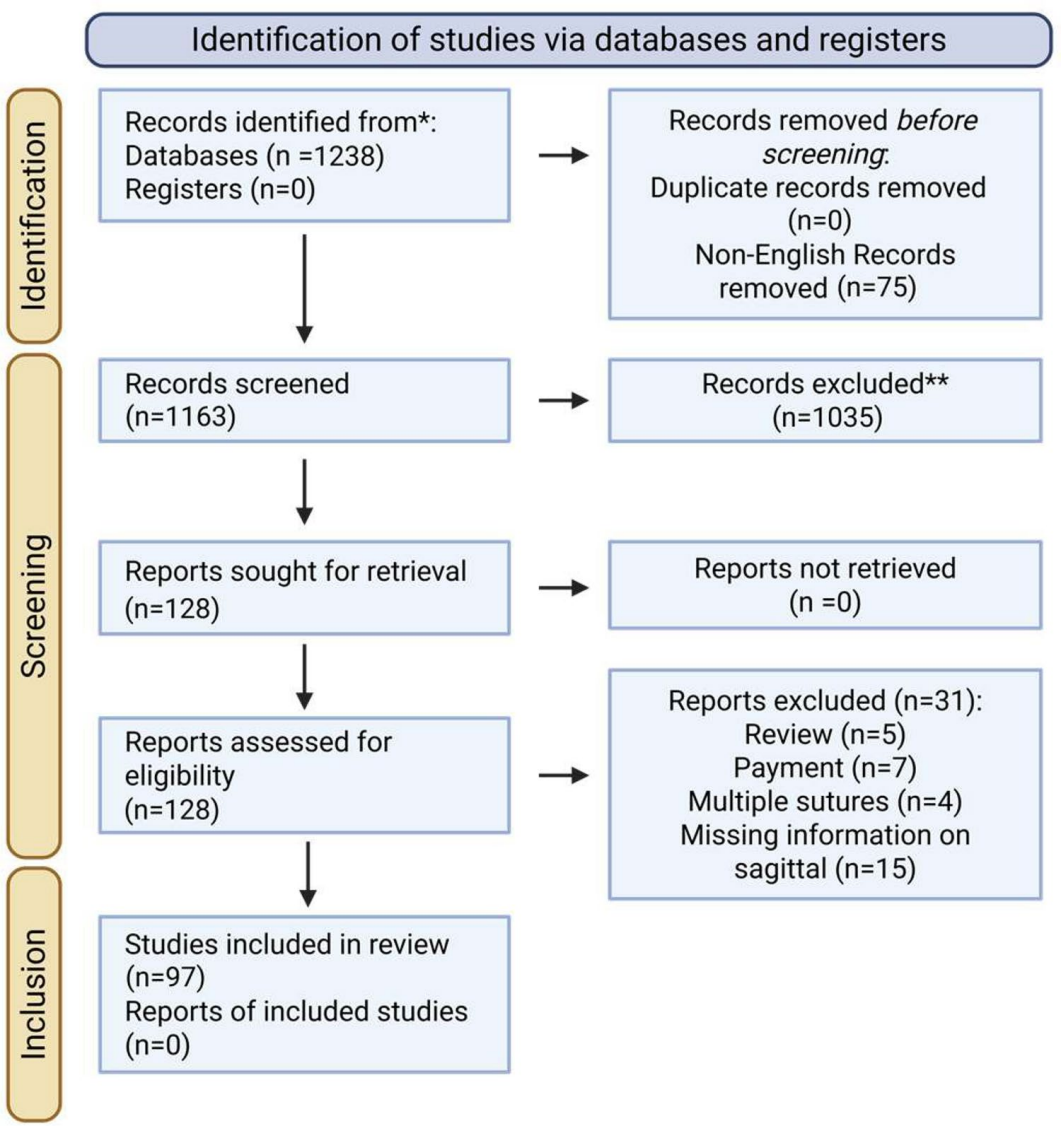
PRISMA flow diagram, illustrating the various steps of the review process, including a detailed account of the number of records passing each phase

A total of 1238 articles were initially identified through the PubMed search. As shown in Fig. 1, only 128 studies were considered sufficiently relevant after the preliminary screening of titles and abstracts. Full-text assessment then led to a final selection of 97 articles that met the criteria of this review, which focuses on the contribution of genetic and environmental factors to the development of sagittal synostosis. Most excluded studies addressed syndromic forms of craniosynostoses, multiple suture craniosynostoses, or non-sagittal synostoses and were therefore not pertinent to the aim of comparing the two etiological domains in sCS. The entire selection process is summarized in Fig. 1.

### Study characteristics

The 97 records passing full-text review included 25,877 patients, among whom 11,086 were diagnosed with sagittal synostosis (42.8%).

A pathogenic germline mutation was reported for 259 patients (12.6%) out of 2060 (75.9%) sequenced and genetically tested patients from 2636 identified patients with sCS.

Data extracted from the 96 included publications are presented in Table 4.

**Table 4:** Characteristics of included studies. Author, journal, study type, and sequencing methodology for the 96 included records are listed. The total number of patients, the number of genetically tested patients with sSC, and the detected variants for each paper are also presented. Environmental determinants, when present in the studies, are also reported. The total number of patients in each of these groups is calculated. *WES*, whole exome sequencing; *DHPLC*, denaturing high-performance liquid chromatography; *MLPA*, multiplex ligation-dependent probe amplification; *TDT*, transmission disequilibrium test; *FISH*, fluorescence in situ hybridization; *ChIP*, chromatin immunoprecipitation, sequencing; *CGH*, comparative genomic hybridization; *OR*, odds ratio; *aPR*, adjusted prevalence ratio; *sCS*, sagittal craniosynostosis; *mCS*, metopic craniosynostosis; *cCS*, coronal craniosynostosis; *lCS*, lambdoid craniosynostosis; *multiCS*, multiple suture craniosynostosis; *SNV*, single nucleotide variant; *SV*, structural variants; *CNV*, copy number variant; *VUS*, variant of uncertain significance; *FGFR −1, −2, −3*, fibroblast growth factor receptor −1, −2, −3; *IHH*, Indian Hedgehog; *BMP −2, −4*, bone morphogenetic protein −2, −4; *BMPER*, BMP binding endothelial regulator; *RUNX2*, runt-related transcription factor 2; *TWIST1*, twist family BHLH transcription factor 1; *TCF12*, transcription factor 12; *ERF*, ETS2 repressor factor; *KRAS*, Kirsten rat sarcoma viral oncogene homolog; *BRAF*, B-Raf proto-oncogene; *PTPN11*, protein tyrosine phosphatase non-receptor type 11; *SIX1*, SIX homeobox 1; *SALL1*, spalt-like transcription factor 1; *SOX6*, SRY-box transcription factor 6; *FOXP2*, Forkhead Box P2; *TFAP2B*, transcription factor AP-2 beta; *GLI2*, GLI family zinc finger 2; *SMAD6*, SMAD family member 6; *TGFBR2*, transforming growth factor beta receptor 2; *NOTCH −1, −2*, notch receptor 1/2; *AXIN1/2*, Axin1/2; *SPRY1/4*, sprouty RTK signalling antagonist 1/4; *ALPL*, alkaline phosphatase; *FBN1*, fibrillin 1; *COL11A1*, collagen type XI alpha 1 chain; *THBS1*, thrombospondin 1; *FREM1*, FRAS1-related extracellular matrix protein 1; *SCARF2*, scavenger receptor class F member 2;* IFT −122, −140*, intraflagellar transport −122, −140; *RAB23*, member RAS oncogene family; *SH3PXD2B*, SH3 and PX Domains 2B; *FLNA*, Filamin A; *ACTB*, beta actin; *PHEX*, phosphate regulating endopeptidase homolog X-linked; *IGF1R*, insulin like growth factor 1 receptor; *IL11RA*, interleukin 11 receptor subunit alpha; *IDUA*, alpha-L-iduronidase; *ACADM*, Acyl-CoA dehydrogenase medium chain; *IVD*, isovaleryl-CoA dehydrogenase; *KMT2D*, lysine methyltransferase 2D; *KAT6B*, lysine acetyltransferase 6B; *PHF17*, PHD finger protein 17; *H2AFV*, H2A histone family member V; *RECQL4*, RecQ like helicase 4; *HUWE1*, HECT, UBA and WWE domain containing E3 ubiquitin protein ligase 1; *ALX4*, ALX homeobox 4; *EFNA4*, ephrin A4; *TRPV4*, transient receptor potential cation channel subfamily V member 4; *MEGF8*, multiple EGF like domains 8; *PRKCE*, protein kinase C epsilon; *MAP4K4*, mitogen-activated protein kinase kinase kinase kinase 4; *KIAA −1109, −1211*, KIAA −109, −1211 protein; *ADAD1*, adenosine deaminase domain containing 1; *IL2/21*, interleukin 2/21; *BBS2/9/12*, Bardet-Biedl syndrome 2/9/12; *FGF −2, −9*, fibroblast growth factor −2, −9; *NUDT6*, nudix hydrolase 6; *SPATA5*, spermatogenesis-associated 5; *FAT4*, FAT atypical cadherin 4; *INTU*, inturned planar cell polarity protein; *SLC25A31*, solute carrier family 25 member 31; *PLK4*, polo-like kinase 4; *MFSD8*, major facilitator superfamily domain containing 8; *PGRMC2*, progesterone receptor membrane component 2; *SCLT1*, sodium channel and clathrin linker 1; *CDH −8, −10*, cadherin −8, −10; *TTC12*, tetratricopeptide repeat domain 12; *CATSPER4*, cation channel sperm-associated 4; *CCBL2*, cysteine conjugate beta-lyase 2; *RASAL2*, RAS protein activator-like 2; *MARCO*, macrophage receptor with collagenous structure; *SLC4A3*, solute carrier family 4 member 3; *EDEM1*, ER degradation enhancing alpha-mannosidase like protein 1; *SI*, sucrase-isomaltase; *ASB5*, ankyrin repeat and SOCS box containing 5; *NME5*, NME/NM23 family member 5; *TENM2*, teneurin transmembrane protein 2; *AGAP3*, ArfGAP with GTPase domain, ankyrin repeat and PH domain 3; *DCAF13*, DDB1 and CUL4-associated factor 13; *NAA25*, N-alpha-acetyltransferase 25; *GPRC5A*, G protein-coupled receptor class C group 5 member A; *MESP1*, mesoderm posterior BHLH transcription factor 1; *TBCB*, tubulin folding cofactor B; *HELZ2*, helicase with zinc finger 2; *CSF2RB*, colony stimulating factor 2 receptor beta common subunit; *CASK*, calcium/calmodulin dependent serine protein kinase; *RARA*, retinoic acid receptor alpha; *SON*, SON DNA binding protein; *NAALADL2*, N-acetylated alpha-linked acidic dipeptidase like 2; *FBLN2*, fibulin 2; *TMEM43*, transmembrane protein 43; *SEMA5A*, semaphorin 5 A; *FASTKD3*, FAST kinase domains 3; *DPP10*, dipeptidyl peptidase-like 10; *PARK2*, Parkin RBR E3 ubiquitin protein ligase; *PACRG*, Parkin co-regulated gene; *RERGL*, RERG like; *TMC1*, transmembrane channel-like 1; *NFIA*, nuclear factor I A; *DPH1*, diphthamide biosynthesis 1

Title of study	First author	Journal (year)	Study type	Sequencing methodology	Controls	Total patients	N. sagittal synostosis	Variants detected	Environmental determinants	Country of the study cohort	Reported males	Reported female
[40]	Accogli	*American Journal of medical Genetics *(2015)	Case report	WES and Array-CGH		2	2	Two SNV on *FGFR3* and deletion of 601.5 Kb at band q26.32 on chromosome 3 gene, candidate gene- *NAALADL2*	Not reported	Italy	2	
[41]	Alghamdi M	*Frontiers in Paediatrics *(2021)	Retrospective cohort study	WES in 22/28 patients		28	4	Likely pathogenic de novo variant in *TCF12*	Not reported	Arabia Saudita	2	2
[42]	Anderson	*Journal of craniofacial surgery *(2007)	Cohort study	DHPLC + WES		8	5	One synonymous polymorphism on *FGFR3*	Not reported	Australia	2	3
[23]	Anderson	*Journal of Clinical Neuroscience *(2007)	Cohort study	Targeted genetic testing for *FGFR* and *TWIST* genes in a subset of patients		41	41	Synonymous polymorphism on *FGFR3* identified in 6/41 patients	Not reported	Australia	26	15
[43]	Arenas	*Journal of Pediatric Endocrinology and Metabolism *(2021)	Retrospective cohort study	*PHEX* gene mutation analysis and molecular testing		96	19	*PHEX* mutations or indels were detected in 14 patients affected by hereditary hypophosphatemic rickets with sagittal suture involvement	Not reported	Argentina		
[44]	Barba M	*Bone *(2018)	Functional molecular study on human surgical samples	Whole transcript profiling; qPCR; *FGFR1–3* and *TWIST1* targeted Sanger sequencing		16	11	Overexpression of specific *BBS9* splice isoforms in fused sagittal/metopic sutures	Not reported	Italy	8	3
[45]	Barik	*Journal of Paediatric Neurosciences *(2013)	Case-control study	Sanger sequencing	120	50	18	No pathogenic *FGFR2* variants	Not found association with advanced parental age	India		
[46]	Barroso	*American Journal of Medical Genetics Part A *(2015)	Case study	Array-CGH + MLPA + Sanger sequencing		2	1	Duplication occurring at upstream regulatory elements of *IHH* (2q35)	Not reported	Spain	1	
[47]	Bashir	*American Journal of Medical Genetics Part A *(2017)	Case report	WES		2	2	Two SV occurring on *KAT6B*	Not reported	Canada	2	
[48]	Baumann	*Human Mutation *(2020)	Case series (4 patients)	WES		4	1	One patient found having a two-nucleotide deletion in *ACTB*	Not reported	USA (Miami)	1	
[49]	Bendon	*BMC Medical Genetics* (2012)	Case report	SNP genotyping + targeted PCR and breakpoint sequencing of *SH3PXD2B*		3	2	*SH3PXD2B*: homozygous deletion of exon 13 (loss of function)	Not reported	UK (Oxford)	1	1
[50]	Bessenyei	*American Journal of Medical Genetics Part A* (2015)	Prospective study	Not reported		200	120	Not reported	Male ratio 3.36, twin gestation ratio 3.06 (mCS 3.44), high incidence of sCS 68%, can be correlated with 26% of women smoking	Hungary	77	23
[51]	Bochukova	*Journal of Medical Genetics* (2010)	Targeted mutation screening	Targeted DNA sequencing for all common mutation *FGFR2, FGFR3, TWIST1*		40	10	No pathogenic variants detected in non-syndromic sagittal synostosis cases; used as genetically negative comparator group	Not reported	UK (Oxford)		
[2]	Boulet	*American Journal of Medical Genetics Part A* (2008)	Retrospective cohort study	Not reported		256	100	Not reported	Maternal age 35–44 ratio 2.32, twins/+3.89 ratio, male ratio 2.48, high weight birth < 4000 g 2.91 ratio	USA (Atlanta)	67	26
[52]	Bradley	*Epidemiology* (1995)	Population-based case-control study	Not reported	291	212	69	Not reported	No increased risk OR 0.9 for paternal occupation on forestry and agriculture	USA (Colorado)		
[53]	Butzellar	*Journal of craniofacial surgery* (2009)	Prospective study	Not reported		30	30	*FGFR2* mutation	Male ratio 2.11	Netherlands	21	9
[54]	Calpena	*Journal of Medical Genetics* (2022)	Cohort study	WES, WGS		1629	3	*SIX1* missense and LoF SNV	Not reported	UK, USA, Brazil		
[31]	Carmichael	*Clinical and molecular teratology* (2007)	Population-based case-control study	Not reported	5008	531	277	Not reported	Moderate increased in the 2nd and 3rd trimester if more than 15 cigarette 1.8 and 1.9 ratio	USA		
[55]	Carmichael	*Birth Defects Research Part A: Clinical and Molecular Teratology* (2010)	Population-based case-control study	Not reported	6789	815	438	Not reported	No intake of supplement OR 1.4 Higher maternal dietary intake of riboflavin (OR 0.5), vitamin B6 (OR 0.4), vitamin E (OR 0.6), and vitamin C (OR 0.7) associated with reduced risk of sagittal craniosynostosis OR 0.92; no association with folic-acid–containing supplements OR 0.9	USA		
[56]	Carmichael	*American Journal of Medical Genetics Part A* (2015)	Population-based case-control study	Not reported	8494	1067	566	Not reported	Maternal factors associated with thyroid dysfunction: fertility medications/procedures (↑ risk, lower than other sutures), younger maternal age (↓ risk OR 0.6), black or other race-ethnicity (↓ risk OR 0.2/0.4/0.6), alcohol consumption (↓ risk (OR 0.8)); maternal thyroid disease itself showed modest association (OR 1.3)	USA		
[57]	Chesler	*Child’s Nervous System* (2018)	Case report	CGH and WES		3	2	No pathogenic mutation detected	Not reported	USA	2	
[25]	Clarke	*American Journal of Medical Genetics Part A* (2018)	Cohort study	RNA sequencing		397	201	Pathogenic/likely pathogenic variants: *SCARF2, TGFBR2, IFT122* and ALPL (p.Thr371Ile) Recurrent VUS: *FLNA*, *KMT2D*, *RECQL4, IGF1R, HUWE1, IDUA, IL11RA, MEGF8, TCF12, FBN1, SH3PXD2B*	Not reported	USA	152	49
[58]	Cuellar	*Bone* (2020)	Study control population	Targeted PCR amplification of the *RUNX2* coding regions + Sanger sequencing		461	270	*RUNX2* mutations	Not reported	USA		
[59]	Cunningham	*American Journal of Medical Genetics Part A* (2011)	Multicenter candidate-gene resequencing cohort study	Sequencing of 27 candidate genes		186	94	*IGF1R* missense variants	Not reported	USA		
[60]	Currarino	*Paediatric radiology* (2007)	Cohort study	Not reported		28	13	Not reported	Not reported	USA	6	7
[61]	Davis	*Global Paediatric Health* (2019)	Case series	Sanger sequencing, WES		5	5	Pathogenic SNV found on *KRAS, BRAF* and *PTPN11*	Not reported	USA	4	1
[62]	Di Rocco	*European journal of human genetics* (2023)	genetic cohort study	NGS (31 panel genes)	101	58	58	Likely pathogenic variants on *SMAD6, FGFR2, TWIST1, ALX4* and *TCF12*	Male ratio 1.9	France	38	20
[63]	Farooq	*Journal of craniofacial surgery* (2020)	Case study	Micro arrays		8	3	One SNV on *ERF*	Maternal history of smoking, breech presentation	UK	1	2
[64]	Fernández-Jaén	*Child’s Nervous System* (2014)	Case report	Array CGH		1	1	De novo deletion at 4q27 involving 18 possible genes (*KIAA1109, ADAD1, IL2, IL21, BBS12, FGF2, NUDT6, SPATA5, SPRY1, FAT4, INTU, SLC25A31, PLK4, MFSD8, PGRMC2, PHF17, SCLT1 e CDH10*)	Not reported	Spain	1	
[65]	Fisher	*Birth Defects Research* (2024)	Population-based case-control study	Not reported	11,216	1432	829	Not reported	No correlation with alcohol	USA	619	210
[66]	Fonteles	*Brazilian Journal of Medical and Biological Research* (2021)	Cross-sectional genetic association study (non-syndromic craniosynostosis cohort)	Sanger sequencing		101	52	SNV found on *ALX4* and *EFNA4*	Not reported	USA		
[67]	Gayden	*Journal of neurosurgery *(2023)	Family-based genetic study	WES		4	1	Mutation found on TRPV4	Not reported	Canada		
[68]	Graham	*American Journal of Medical Genetics Part A* (1998)	Case report	Short Tandem Repeat genotyping methodology		6	3	Linkage analysis in three families with FG syndrome resulted in a broad localization ranging from the AR locus in Xq12 to the DXS1210 locus in Xq23	Not reported	USA	3	
[12]	Greenwood	*Genetics in Medicine *(2014)	Large multicentre familial aggregation and phenotype study (International Craniosynostosis Consortium)	Sanger sequencing		660	301	No pathogenic variants detected	Not reported	USA	223	78
[69]	Honein	*Teratology *(2000)	Retrospective study	Not reported		44	27	Not reported	Small number of sample but any smoking OR ratio 1.71 moderate smoking in the first 3 months of pregnancy has odds ratio with sagittal of 5.12 and heavy smoking OR 2.03	USA		
[70]	Hove	*Clinical Dysmorphology *(2026)	Twin study	Not reported		2	1	Not reported	Monozygotic twins, with same genes have different suture synostosis	Denmark	2	
[71]	Ittleman	*American Journal of Medical Genetics Part A *(2017)	Cohort study	SNP array		139	1	Found a 2q37 deletion	Not reported	USA (Arkansas)		
[72]	Jenkins	*American Journal of Human Genetics *(2007)	Multicentre genetic study of syndromic craniosynostosis	Genome-wide SNP genotyping; linkage analysis; targeted Sanger sequencing of *RAB23* (all exons and splice sites); MLPA to exclude deletions		17	2	Biallelic loss-of-function mutations in *RAB23*	Not reported	Denmark, USA	1	1
[73]	Johnson	*American Journal of Human Genetics *(1998)	Cohort study	Sanger sequencing		53	5	*TWIST* mutation screening, no mutations found on sagittal patients	Not reported	UK and other		
[74]	Junaid	*The Cleft Palate Craniofacial Journal *(2022)	Population-based case-control study	Not reported		5854	2039	Not reported	Male OR 1.75	Australia	1472	567
[75]	Justice	*American Journal of Medical Genetics Part A *(2017)	Human genetics + molecular functional study	Molecular in vitro assays (luciferase reporter assay) In vivo functional validation (zebrafish transgenesis)		1	1	Non-coding regulatory SNP rs1884302 (C risk allele) downstream of non-coding region of *BMP2* shows increased enhancer activity and altered spatial expression in vivo; supports *BMP2* overexpression as pathogenic mechanism	Not reported	Not reported		
[76]	Justice CM	*Nature Genetics *(2012)	Genome-wide association study (GWAS)	Genome-wide SNP genotyping; TDT analysis in case-parent trios; replication in independent case-control cohort	548	302	302	Common risk SNPs found near *BMP2* (20p12.3; lead SNP rs1884302) and within *BBS9* (7p14.3; lead SNP rs10262453); no rare pathogenic coding variants identified	Not reported	USA		
[15]	Kallen	*Teratology *(1999)	Retrospective cohort study	Not reported		304	145	Not reported	Maternal smoking associate with sagittal with ratio of 1.48, higher than the other sutures, parity 4 + OR 2.0, education level > 15 y OR 1.6 male OR 2.8	Sweden	108	37
[77]	Klopocki	*American Journal of Human Genetics *(2011)	CNV-based genetic association study	Array CGH; breakpoint PCR and Sanger sequencing; qPCR for segregation; LacZ reporter assays in transgenic mice		77	1	Microduplications at 2q35 involving noncoding regulatory elements upstream of *IHH* (48–59 kb); duplications overlap conserved noncoding enhancers → *IHH* misexpression/overexpression; CNVs segregate with craniosynostosis and Philadelphia type	Not reported	Germany		
[78]	Körberg	*BMC Medical Genetics *(2020)	Case report	WES		2	1	Frameshift mutation on *ERF*	Not reported	Sweden		1
[79]	Kratz	*American Journal of Human Genetics *(2009)	Clinical report	WES		2	1	One SNV found on *KRAS*	Not reported	Europe	1	
[3]	Lajeunie	*American Journal of Medical Genetics *(1996)	Retrospective cohort with segregation (family-based) analysis	Statistical genetic segregation analysis		1408	561	Not reported	Male ratio 3.5:1, the study affirms that the defect was transmitted as a dominant disorder with 38% penetrance	France	436	125
[80]	Lapehn	*International Journal of Molecular Sciences *(2023)	Case-control study	RNA sequencing		388	196	Not reported	Male OR 1.46	USA	148	48
[81]	Lee	*Child’s nervous System *(2019)	Cohort study	PCR amplification		1	1	Large deletion comprising exons 1–3 of *PHEX* found in XLHR patient affected by sagittal synostosis	Not reported	Korea	1	
[82]	Lee	*Journal of craniofacial Surgery *(2012)	Case report	Not reported		522	246	Not reported	Male ratio OR 2.71, Macrosomia (> 4000 g) OR 1.61 and large gestational weight risk OR 1.72, high maternal age risk ratio 2.01 (mCS 3.0) previous live birth 2+ OR 1.39 (cCS OR 1.82)	Australia (Victoria)	150	55
[83]	Lee	*Genetics in medicine *(2018)	Retrospective cohort study	Sanger sequencing		309	18	Deletion causing early stop codon on *ERF*	Not reported	Australia		
[84]	Lugli	*European Journal of Medical Genetics *(2022)	Case report	NGS		2	2	One patient with SNV on *SALL*1	Not reported	Italy	2	
[85]	Maldziene	*Clinical Dysmorphology *(2019)	Case report	Chromosomal microarray analysis		1	1	16p13.11-p12.3 microdeletion	Not reported	Lithuania		1
[86]	McGillivray	*Journal of Medical Genetics (2005)*	Clinical and molecular family study	Sanger sequencing		11	7	Mutation found on *FGFR2*	Male ratio 2.6:1	Australia	8	3
[87]	Mefford	*The American Journal of Medical Genetics—Part A *(2010)	Cohort study	Array CGH and FISH		186	7	Various large duplication and deletion found, possible affected genes (FBLN2, TMEM43, SEMA5A, FASTKD3 DPP10 PARK2, PACRG PRKCE, RERGL, and TMC1)	Not reported	USA		
[88]	Miller	*Journal of Medical Genetics* (2016)	Cohort study	WES and WGS		40	3	No mutation found on sagittal patients	Not reported	Europe (8 South Asia, 1 Middle East, 1 African)		
[89]	O'Brien	*Birth Defects Research Part A: Clinical and Molecular Teratology* (2017)	Population-based case-control study	Not reported	2993	316	163	Not reported	Maternal occupational exposure to polycyclic aromatic hydrocarbons (PAHs) during the early months of pregnancy and risk of craniosynostosis among offspring (OR 1.44) adjusted to sagittal OR 1.76 but non statistically significant	USA		
[90]	Ohishi	*The American Journal of Medical Genetics—Part A *(2017)	Case series	Sanger sequencing		11	1	Mutation on *FGFR2*	Not reported	Japan		1
[91]	Park	*Bone *(2016)	Observational, case-control, functional genomics study	No sequencing (Genomic transcriptomic profiling for gene expression analysis)		249	100	Sagittal synostosis in females may be distinct from that of males as evidenced by our identification of a unique set of genes expressed in osteoblast cell lines derived from females with sagittal synostosis. The identification of genes whose expression is correlated with differentiation and is sex specific suggests that these transcripts may play a role in the male predominance of sagittal and metopic craniosynostosis and furthermore, that females with sagittal craniosynostosis may have different genetic risk profiles	Not reported	USA		
[9]	Passos-Bueno	*American Journal of Medical Genetics *(2002)	Case report	DNA sequencing		3	2	Not reported	Not reported	Brazil	2	
[92]	Priolo	*American Journal of Medical Genetics *(2001)	Case report	Sanger sequencing		1	1	Not reported	Pregnancy complications (maternal hypertension, preeclampsia)	Italy	1	
[93]	Rasmussen	*Obstetrics & Gynecology *(2007)	Population-based case-control study	Not reported	4094	431	212	Not reported	Mothers of infants with craniosynostosis were more likely than mothers of control infants to be older, have a higher level of education, be of non-Hispanic white ethnicity, have a BMI in the obese range, to be multigravid, and to have pregestational diabetes, sCS + thyroid diseases ORs 2.11 (cCS OR 3.32, multiCS OR 8.73)	USA		
[94]	Rodriguez-Zabala	*Human mutation *(2017)	Case report	NGS		2	2	Two mutations found in heterozygosis on *FGF9*	Not reported	Spain	1	1
[95]	Rothenbuhler	*The Journal of Bone and Mineral Research *(2019)	Cohort study	Genetic analysis		44	23	Inactivating *PHEX* mutations identified in XLHR patients, including patients with sagittal craniosynostosis	Not reported	France		
[29]	Sanchez-Lara	*American Journal of Medical Genetics *(2011)	Population-based, multi-site case-control study	Not reported	5928	670	357	Not reported	Plurality Twins/+ and fertility treatment 1.6 OR risk preterm gestation risk OR 1.7	USA		
[96]	Schraw	*Birth Defects Research *(2020)	Population-based retrospective cohort study	Not reported		2111	1011	Not reported	Male aPR 2.88, increase for mother that lives US-Mexico borders (OR 1.29) (cCS OR 1.65), increased risk for > 1 previous births OR 1.26, preterm < 37 gestation OR 1.22 (mCS OR 1.58), pre pregnancy obesity associated with increased risk, offspring of non-Hispanic black and Hispanic women had lower risk of CS Birth prevalence of sCS increased 1.35 to 2.03 per 10 000 live birth from 1999 to 2014	USA (Texas)	760	251
[97]	Selber	*Plastic and reconstructive Surgery *(2008)	Cohort study	Not reported		854	583	Not reported	Male ratio 3.2:1	USA	444	139
[98]	Seto	*American Journal of Medical Genetics *(2007)	Cohort study	Sanger sequencing		164	83	*Mutations found on TWIST1*, *FGFR2*, and FGFR1	Male ratio 3.6:1	USA	65	18
[99]	Sewda	*Pediatrics Research *(2019)	Cohort study	NGS		59	40	Mutations found on *ALX4, ADCK1, ALPL, RECQL4, SH3PXD2B, BMPER, FREM1, NOTCH1, NOTCH2, PDILT*, and* TGFBR2*	Not reported	USA, Spain	27	13
[100]	Sharma	*Cleft Palate Craniofacial Journal *(2012)	Case report	Sanger sequencing		1	1	One SNV on *FGFR2*	Not reported	UK	1	
[101]	Sharova	*Genes *(2023)	Case series	WES		3	1	Mutation on canonical splicing donor site of *IFT140*	Not reported	Russia	1	
[102]	Singer	*American Journal of Medical Genetics *(1999)	Population-based case-control study	Not reported	522	170	70	Not reported	Male ratio OR 2.62 Maternal age 35+ OR 2.34, paternal age 40+ OR 2.11 (lCS 5.09), preterm gestation < 37 weeks OR 2.41 (lCS 3.09), Very low birth weight < 2500 g OR 2.32 (lCS OR 3.98), birth weight 3500–3999 g OR 1.98 Breech presentation OR 2.39	Western Australia	56	14
[103]	Somolan	*Maedica *(2002)	Retrospective cohort study	Not reported		35	26	Not reported	Male ratio 2.5:1, more rural area patient	Netherlands		
[104]	Stamper	*PlosOne *(2011)	Case-control study	No sequencing (Microarrays for gene expression analysis)		199	100	Differentially expressed genes in sagittal cases, including *FGF7*, *VCAM1*, *SFRP4*, and sagittal-specific expression signatures involving ECM and focal adhesion pathways	Not reported	USA	77	23
[105]	Timberlake	*Journal of Neurosurgical paediatrics *(2024)	Observational study	WES		6	3	Mutations on *AXIN2*	Not reported	International	3	
[106]	Timberlake	*Proceedings of the National Academy of Sciences of the United States of America *(2019)	Observational genetic study	WES		12	3	Mutations on *TFAP2B, GLI2*, and* CTNNA1*	Not reported	International	3	
[107]	Timberlake	*eLife *(2016)	Prospective and retrospective cohort study	WES		191	113	Mutations on *SMAD6, SPRY4, SPRY1, TTC12, ACADM, CATSPER4, CCBL2, COL11A1, RASAL2, MARCO, SLC4A3, EDEM1, SI, KIAA1211, ASB5, NME5, TENM2, AGAP3, H2AFV, DCAF13, NAA25, GPRC5A, MESP1, IVD, THBS1, TBCB, HELZ2*, and* CSF2RB*	Not reported	International		
[108]	Timberlake	*American Journal of Human Genetics *(2023)	Trio-based gene-discovery study	WES		526	/	Mutations on *ADNP CASK, RARA*, and* SON*	Not reported	USA		
[109]	Tolchin	*American Journal of Human Genetics *(2020)	Cohort study	WES, WGS		19	1	SNV on *SOX6*	Not reported	Europe, USA	1	
[110]	Tonne	*European Journal of Human Genetics *(2020)	Cohort study	WES, Sanger sequencing, MLPA, array CGH		10	3	Mutations on *EXTL3* and *FOXP2*	Not reported	Norway	2	
[111]	Ueda	*European Journal of Medical Genetics *(2015)	Case series	FISH		8	6	All reported patients with 7q11.23 deletion	Not reported	Japan	2	4
[112]	Ueda	*American Journal of Medical Genetics *(2017)	Case series	Sanger sequencing		9	5	Mutations on *BRAF* and *PTPN11*	Not reported	Japan	3	2
[113]	Walton	*Journal of Anatomy *(2024)	Case-control study	PCR		389	225	Small deletion on *RUNX2*	Not reported	UK		
[114]	Weng	*Environment International *(2024)	Case-control study	RNA-seq analysis + ChIP		244	164	Not reported	Nickel dose effect hypomethylation of FGFR2 and increases binding to Sp1, promoting osteogenic differentiation	China		
[115]	Wentz	*Eur Child Adolesc Psychiatry *(2014)	Case report	Microarray analysis and DNA sequencing		2	2	Duplication Xq13.1–q21.1	Not reported	Sweden	2	
[8]	Wilkie	*Pediatrics *(2013)	Cohort study	CGH		326	117	Mutations on *FGFR2* and *FGFR3*	Not reported	UK		
[116]	Xu	*Gene *(2018)	Case report	WES		9	1	Pathogenic deletion on FGFR2	Not reported	China	1	
[117]	Xu	*Environment International *(2021)	Case series	Not reported		259	174	Not reported	Male ratio 2:1, nickel, tin, and chromium dose effect	China	118	56
[118]	Xu	*Hereditas *(2021)	Case–control study	WES		1	1	Mutation on *AXIN2 in heterozygosis*	Persistent breech presentation, intrauterine growth restriction, twin pregnancy, prematurity, maternal metabolic/thyroid alterations	China	0	1
[119]	Yagnik	*Human mutation *(2013)	Cohort study	Sanger sequencing		203	197	Different *ALX4* gain-of-function variants	Not reported	International		
[120]	Ye	*Plastic and reconstructive Surgery *(2017)	Case-control study	DNA Sanger sequencing		93	93	Found: insertion in *FGFR1*, pathogenic SNV in *TWIST1*, and VUS on* RAB23*	Not reported	USA (Iowa + New York)		
[121]	Yilmaz	*American Journal of Medical Genetics *(2018)	Case report	DDR panel v2 of Ion AmpliSeq and Sanger sequencing		1	1	Small deletion on *AXIN2*	Not reported	Turkey		
[122]	Yoon	*Clinical Genetics *(2024)	Case report	WES		1	1	Missense SNV on MAP4K4	Not reported	Korea	1	
[123]	Yoon	*Neurosurgery *(2020)	Cohort study	NGS		110	36	Mutations found on *ERF*, *ALPL*, *FBN1*, *ALX4*, *FGFR3*, and *BMP4* Large deletions on 1p31.3 (candidate gene: *NFIA*) and 17p13.3 (Candidate gene: *DPH1*)	Not reported	Korea		
[124]	Yu	*Medicine *(Baltimore)(2017)	Case report	WES		1	1	Microduplications at 8p11.22–q12.1 and 16q11.2–q21	Not reported	China	1	
[26]	Zeiger	*The Journal of Craniofacial Surgery *(2002)	Population-based case-control study	Targeted PCR-based mutation analysis	182	42	42	No pathogenic mutation detected	Male ratio (4.5:1). Control/case higher education (mom OR = 3.3, dad OR = 4.4) no correlation with smoking	USA (Washington)	34	8
[125]	Zollino	*American Journal of Medical Genetics *(1999)	Case report	R-banding and FISH;		2	1	Partial duplication of distal 15q (15q25.1–qter trisomy) due to unbalanced familial translocation t(13;15)	Not reported	Italy		1

Studies that demonstrated the involvement of genetic factors were 66, of which 31 were reported as case study, case report, case series; 34 were reported as observational retrospective, cohort, case control; and 1 study was an experimental functional study.

Studies with a focus on environmental engagement were 25, out of which 2 were reported as case report, 1 case series, 3 as case-control study, 12 as population-based case-control study, 5 as retrospective study, 1 as prospective study, and 1 as twin study.

Seven of these studies presented both genetic and environmental factor out of which 4 case-control study, 1 cohort study, 1 clinical and molecular family study, and 1 prospective study.

### Synthesis of data

The extracted data were grouped with focus on genomic location and frequency and environmental factors. Germline genetic testing, performed in sCS patients, highlights the variants listed in Table 5.

**Table 5:** Sagittal craniosynostosis (sCS)-associated genes. Overview of genes in which pathogenic variants in one or more than one patient with syndromic and non-syndromic sCS have been reported. The genomic location and name of each gene with one or more than one detected variant are tabulated. The patients with detected variants for each gene are then listed, along with the mutation reference (if specified in the article) and the resulting protein change, if applicable

N° patients	Chromosome	Gene	Single nucleotide variant (SNV) or structural variant (SV)	Pathogenic variant	Assembly nomenclature/reference	Protein chance
1	1p31.1	*ACADM*	Damaging missense (de novo)		NM_001286044	Ala103Thr
1	7p22.1	*ACTB*	SV	c.890_891delCA	NM_001101.4 and NG_007992.1	Thr297Serfs*37
1	8p21.1	*ADCK1*	SNV	c.467C>T	NM_020421	Thr156Met
1	3q27	*ADPN*				
1	7q21.2	*AGAP3*	Damaging missense (de novo)		NM_001281300	Arg185Trp
1	1p36.12	*ALPL*	SNV	c.212G>C	NM_000478	Arg71Pro
1	1p36.12	*ALPL*	VUS	c.961C>T	NM_000478.5	Arg321Trp
1	1p36.12	*ALPL*	De novo SNV	c.529G>A	NM_000478.5	Ala177Thr
1	1p36.12	*ALPL*	Maternal inherited SNV	c.668G>A	NM_000478.5	Arg223Gln
1	1p36.12	*ALPL*	SNV	c.1112C>T	NM_000478.5	Thr371Ile
n/a	11p11.2	*ALX4*	Likely pathogenic SNV	c.917C>T	rs149897209	Pro306Leu
n/a	11p11.2	*ALX4*	Likely pathogenic SNV	c.631A>G		Lys211Glu
n/a	11p11.2	*ALX4*	Likely pathogenic SNV	c.19G>T		Val7Phe
3	11p11.2	*ALX4*	SNV	c.799G>A		Ala267Thr
1	11p11.2	*ALX4*	SNV	c.126G>C	NM_021926	Lys42Asn,
1	11p11.2	*ALX4*	VUS	c.226C>A	NM_021926.3	Pro76Thr
n/a	11p11.2	*ALX4*	Non synonymous SNV	c. 104G>C	rs3824915	Arg35Thr
n/a	11p11.2	*ALX4*	Non synonymous SNV	c. 304G>T	rs12421995	Pro102Ser
*1*	11p11.2	*ALX4*	SNV	c.646C>T	NM_021926.4	p.(Arg216Trp)1
1	14q24.3	*ASB5*	Damaging missense (de novo)		NM_080874	His238Leu
3	16p13.3	*AXIN1*	De novo SNV	c.A965G	NM_181050.2	Glu322Gly
n/a	16p13.3	*AXIN1*	SNV	c.G1235A	NM_181050.2	Arg412Gln
n/a	16p13.3	*AXIN1*	SV	c.A2187-2G IVS10-2A>G	NM_181050.2	
1	8p11.23	*AXIN2*	SV	c.1045_1046delCT	NM_004655.3	Lys349fs*24
1	17q24.1	*AXIN2*	SNV	c.1181G>A	rs200899695	Arg394His
1	7p14.3	*BBS9*	Decreased risk SNP	Minor allele/major allele C/A	rs10262453	
n/a	20p12.3	*BMP2*	Increased risk SNP	Minor allele/major allele C/T	rs1884302	
1	14q22.2	*BMP4*	VUS	c.751C>T	NM_130851.3	His251Tyr
2	7p12.3	*BMPER*	SNV	c.1663C>T	NM_133468	Arg555Trp
n/a	7q34	*BRAF*	SNV	c.1497A>C		Lys499Asn
n/a	7q34	*BRAF*	SNV	c.735A>T		Leu245Phe
1	7q34	*BRAF*	SNV	c.770A>G		Gln257Arg
3	7q34	*BRAF*	SNV	c.1741A>G		Asn581Asp
1	7q34	*BRAF*	SNV	c.755G>C		Arg252Pro
1	Xp11.4	*CASK*			NM_003688.3	
1	1p36.13	*CATSPER4*	Damaging missense (de novo)		NM_198137	Ile289Val
1	20q11.23	*CCBL2*	Damaging missense (de novo)		NM_001008662	Gly260Asp
1	1p21.1	*COL11A1*	Damaging missense (de novo)		NM_080630	Gly1034Ser
1	22q12.3	*CSF2RB*	Damaging missense (de novo)		NM_000395	Gly635Trp
n/a	5q31.2	*CTNNA1*	SV	c.1121_1122insGTCATGGAAGATGAA	NM_001324010	Val374_375insSerTrpLysMetLys
1	17p13.3	*DPH1*	SV	17p13.3 1452 deletion		
1	1p31.3	*NFIA*	SV	1p32-p31 7765 kb deletion	no. 613735	
1	8p11 and 16q11	*FGFR1, BBS2, SALL1, CDH8)*	SV	8p11.22 q12.1 (18Mbp) and 16q11.2 q21 (18Mbp) microduplication		
1	3p25	*FBLN2, TMEM43*	SV	duplication	Chr3: 11.96–15.30 Mb	
1	3q29	*DCAF13*	Nonsense (loss of function) (de novo)		NM_015420	Arg518*
1	2q14	*DPP10*	SV	Duplication	Chr2: 115.97–116.70 Mb	
1	3q27.2	*EDEM1*	Damaging missense (de novo)		NM_014674	Asp188Asn
n/a		*EFNA4*	SNV	C.283A>G		Lys95Glu
1	19q13.2	*ERF*	SV	c.90_99del	NM_006494.2	Trp30Cysfs*44
1	19q13.2	*ERF*	SNV	c.886G>A	NM_006494.3	Gly296Ser
1	12q24.13	*ERF*	SNV	c.202G>C		Gly68Arg
1	19q13.2	*ERF*	Maternal inherited SV	c.985_1027del	NM_006494.3	Arg329Serfs*54
1	19q13.2	*ERF*	SV	c.1201_1202delAA		Lys401Glufs*10
1	8p21.1	*EXTL3*	Likely pathogenic (PM2, PP4 [moderate] PP2, PP3) SNV	c.2392G>A	NM_001440.3	(Val798Met)
1	15q21.1	*FBN1*	VUS	c.6878A>G	NM_000138.4	Asn2293Ser
1	15q21.1	*FBN1*	Likely pathogenic SNP	c.6446A>G	NM_000138.4	Tyr2149Cys
n/a	15q21.1	*FBN1*	SNV	c.7661G>A	NM_000138.4	Arg2554Gln
2	13q12.3	*FGF9*	SNV	c.184A>G	NM_002010.2	Arg62Gly
1	8p11.23	*FGFR1*	SNV	c.936 + 113C>T and c.1508C>T	NM_000604.2 and NM_023107.1	Thr261Met
1	8p11.23	*FGFR1*	Likely pathogenic SV	c.730_731insG	NM_023105	Ala244fs*26
1	10q26.13	*FGFR2*				
1	10q26.13	*FGFR2*	Likely pathogenic SNP	c.1576A → G		Lys526Glu
1	10q26.13.1	*FGFR2*	Pathogenic SV	c.833_834 delinsTT		Cys278Phe
1	10q26.13	*FGFR2*	De novo	c.184T>C		Cys62Arg
1	10q26.13	*FGFR2*	SNV	c.940-57C>T	NM_000141.2	
2	10q26.13	*FGFR2*	Familial SNV (5 other cases)	1032G>A		Ala344Ala
1	10q26.13	*FGFR2*	SNV	c.68C>A	NM_000141.5	p.(Pro23His)
1	10q26.13	*FGFR2*	SNV	c.820G>A	NM_000141.5	p.(Val274Ile)
n/a	10q26.13	*FGFR2*	SNV	c.1646A>C		Asn549Thr
1	4p16.3	*FGFR3*	De novo SNV	1620C>A		Asn540Lys
1	4p16.3	*FGFR3*	SNV	c.882T>C		Asn294Asn
1	4p16.3	*FGFR3*	VUS	c.1730T>C	NM_001163213.1	Leu577Pro
6	4p16.3	*FGFR3*	SNP	294C>T		Asn294Asn
1	4p16.3	*FGFR3*	SNV	c.1138G>A	NM_000142	Gly380Arg
1	4p16.3 + 3q26.32	*FGFR3* + *candidate gene (NAALADL2)*	SNV + SV	c.1138G>C + 3q26.32 601 kb deletion	NM_000142	Gly380Arg
n/a	Xq28	*FLNA*	SNV	c.4625C>T	NM_001456.3	Thr1542Ile
n/a	Xq28	*FLNA*	SNV	c.842C>T	NM_001456.3	Pro281Leu
n/a	Xq28	*FLNA*	SNV	3c.4897C>T	NM_001456	Arg1633Cys
n/a	Xq28	*FLNA*	SNV	c.3348C>A	NM_001456.3	Asp1116Glu
n/a	Xq28	*FLNA*	SNV	c.3755C>T	NM_001456.3	Ala1252Val
1	7q31.1	*FOXP2*	Likely pathogenic (PVS1, PM2, PS4) SV	c.484del	NM_148899.3	(Gln162fs)
1	9p22.3	*FREM1*	SNV	c.1394G>C	NM_144966	Gly465Ala
1	9p22.3	*FREM1*	SNV	c.3819T>A	NM_144966	Asp1273Glu
n/a	2q14.2	*GLI2*	De novo SNV	c.G1651A	NM_005270	Ala551Thr
1	12p13.1	*GPRC5A*	Nonsense (loss of function) (de novo)		NM_003979	Arg272*
3	Xq12-q21	*Good candidate XNP*	Locus inactivation			
1	1q42.12	*H2AFV*	Damaging missense (de novo)		NM_012412	Ala6Pro
1	20q11.21	*HELZ2*	Damaging missense (de novo)		NM_033405	Arg120His
n/a	Xp11.22	*HUWE1*	SNV	c.9800G>A	NM_031407.6	Arg3267His
n/a	4p16.3	*IDUA*	SNV	c.686C>G	NM_000203.4	Pro229Arg
n/a	3q21.1	*IFT122*	SV	c.3624+1G>A	NM_052985.3	
1	10q26.13	*IFT140*	Canonical splicing donor site	c.2767_2768+2de	NM_014714.4: chr16:1575885CACTA>C	
1	15q26.3	*IGF1R*	SNP	FN3 domain	rs45611935	Asn857Ser
1	15q26.3	*IGF1R*	SNP	Recep_L_domain		Arg406His
n/a	15q26.3	*IGF1R*	SNV	c.4058G>A	NM_000875.4	Arg1353His
n/a	2q35	*IHH (upstream regulatory elements)*	SV	Microduplication (31 kb)		
25	9p13.3	*IL11RA*	SNV	c.388T>A	NM_001142784.2	Cys130Ser
1	15q14	*IVD*	Damaging missense (de novo)		NM_002225	Arg53His
n/a	10q22.2	*KAT6B*	SV	c.4205_4206delCT		Ser1402Cysfs*5
2	10q22.2	*KAT6B*	SV	c.4572dupT		Thr1525Tyrfs*16
1	2q31.1	*KIAA1211*	Nonsense (loss of function) (de novo)		NM_020722	Gly17*
n/a	12q13.12	*KMT2D*	SNV	c.7036G>A	NM_003482.3	Gly2346Ser
n/a	12q13.12	*KMT2D*	SNV	c.13780G>C	NM_003482.3	Ala4594Pro
n/a	12q13.12	*KMT2D*	SNV	c.7198C>G	NM_003482.3	Pro2400Ala
n/a	12q13.12	*KMT2D*	SNV	c.9212G>A	NM_003482.3	Arg3071Lys
1	12p12.1	*KRAS*	SNV	c.173C>T		
1	12p12.1	*KRAS*	SNV	c.40G>A		Val14Ile
1	17q24.1	*MAP4K4*	Missense SNV	c.569G>T	NM_001242559	Gly190Val
1	12q15	*MARCO*	Damaging missense (de novo)		NM_006770	Lys201Thr
n/a	19q13.11	*MEGF8*	SNV	c.3685G>A	NM_001410.2	Gly1229Arg
1	15q26.1	*MESP1*	Nonsense (loss of function) (de novo)		NM_018670	Glu104*
1	4q27	*Multiple genes (18): KIAA1109, ADAD1, IL2, IL21, BBS12, FGF2, NUDT6, SPATA5, SPRY1, FAT4, INTU, SLC25A31, PLK4, MFSD8, PGRMC2, PHF17, SCLT1 e CDH10*	SV	11 Mb deletion	chr4: 123094652-−134164491	
1	12q15	*NAA25*	Frameshift (loss of function) (de novo)		NM_024953	Phe359fs*26
1	17q21.32	*NME5*	Frameshift (loss of function) (de novo)		NM_003551	Ile153fs*9
3	9q34.3	*NOTCH1*	SNV	c.2734C>T	NM_017617	Arg912Trp
1	6q26	*PARK2, PACRG*	SV	Deletion	Chr6: 162.84-−163.46 Mb	
2	16p13.11	*PDILT*	SNV	c.1243C>T	NM_174924	Pro415Ser
23	Xp22.11	*PHEX*				
1	Xp22.11	*PHEX*	SV	Large deletion comprising exons 1–3		
7	Xp22.11	*PHEX*	SNV	c.1072A>T	NG_007563.2	Arg358Ter
n/a	Xp22.11	*PHEX*	SNV	c.2069A>C	NG_007563.2	His690Pro
n/a	Xp22.11	*PHEX*	SNV	c.2104C>T	NG_007563.2	Arg702Ter
n/a	Xp22.11	*PHEX*	SV	c.293delT	NG_007563.2	Met98SerfsTer10
n/a	Xp22.11	*PHEX*	SV	c.293delT	NG_007563.2	Met98SerfsTer10
n/a	Xp22.11	*PHEX*	SNV	c.1699C>T	NG_007563.2	Arg567Ter
n/a	Xp22.11	*PHEX*	SNV	c.1586+1G>A	NG_007563.2	
1	2p21	*PRKCE*	SV	Duplication	Chr2: 45.75-−46.15 Mb	
1	12q24.13	*PTPN11*	SNV	c.188A>G		Tyr63Cys
1	12q24.13	*PTPN11*	SNV	c.1471C>G		Pro491Ala
2	12q24.13	*PTPN11*	SNV	c.922A>G		Asn308Asp
2		*RAB23*	SNV	c. 434T>A		Leu145X
1	6p11.2	*RAB23*	VUS	546A>C	NM_016277.5	Glu>Asp
1	17q21.2	*RARA*	SNV	c.865G>A	NM_000964:exon7	Gly289Arg
1	1p21.1	*RASAL2*	Damaging missense (de novo)		NM_004841	Arg571Pro
1	8q24.3	*RECQL4*	SNV	c.2435G>A	NM_004260	Cys812Tyr
1	8q24.3	*RECQL4*	SNV	c.2340G>T	NM_004260	Pro780Pro
1	8q24.3	*RECQL4*	SNV	c.1565G>A	NM_004260	Arg522His
1	8q24.3	*RECQL4*	SNV	c.2237C>T	NM_004260	Ala746Val
1	8q24.3	*RECQL4*	SNV	c.212A>G	NM_004260	Glu71Gly
n/a	8q24.3	*RECQL4*	SNV	c.3430C>T	NM_004260.3	Arg1144Cys
n/a	8q24.3	*RECQL4*	SNV	c.1063C>T	NM_004260.3	Arg355Trp
1	12p12	*RERGL*	SV	deletion	Chr12: 18.12–18.20 Mb	
33	12q24.13	*RUNX2*	SV	c.243_260del	NM_001024630.4:rs11498192	(Ala84_Ala89)del
1	6p21.1	*RUNX2*	SNV	c. 1361 A>G		Tyr454Cys
1	6p21.1	*RUNX2*	SNV	c. 1000 G>A		Asp334Asn*
2	6p21.1	*RUNX2*	SNV	c. 1531 G>A		Gly511Ser*
1	6p21.1	*RUNX2*	SNV	c. 751 C>T		Arg251Cys*
42	6p21.1	*RUNX2*	SV	/		Ala84-Ala89del*
1		*SALL1*	SNV	c.709C>T	NM_002968.3	Gln237*
n/a	22q11.21	*SCARF2*	SNV	c.1999A>C	NM_153334.6	Lys667Gln
n/a	22q11.21	*SCARF2*	SNV	c.1688T>G	NM_153334.6	Val563Gly
1	5p15	*SEMA5A, FASTKD3*	SV	Duplication	Chr5: 7.59-−10.06 Mb	
1	5q35.1	*SH3PXD2B*	SNV	c.970C>T	NM_001017995	Arg324Trp
n/a		*SH3PXD2B*	SV	Homozygous deletion of exon 13 (LoF)		
	5q35.1	*SH3PXD2B*	SNV	c.2276C>G	NM_001017995.2	Pro759Arg
1	3q25.1	*SI*	Damaging missense (de novo)		NM_001041	Ile1329Val
n/a	14q23.1	*SIX1*	SNV	c.40G>C		Val14Leu
n/a	14q23.1	*SIX1*	SNV	c.513G>A		Trp171*
3	14q23.1	*SIX1*	SNV	c.452C>T	NM_005982	Pro151Leu
1	2q12.3	*SLC4A3*	Damaging missense (de novo)		NM_005070	Arg553Cys
1	5q31.2	*SMAD6*	Damaging SNV	c.A916G		Thr306Ala
1	5q31.2	*SMAD6*	Frameshift SV	c.1034delG		Arg345fs*194
1	5q31.2	*SMAD6*	Frameshift SV	c.839_840insT		Arg281fs*13
1	5q31.2	*SMAD6*	Stop SNV	cG1120T		Glu374*
1	15q22.31	*SMAD6*	SNV	c.874+4A>G,	NM_005585.4	
1	15q22.31	*SMAD6*	SNV	c.91+4 A>G	NM_001142861.2	
2	21q22.11	*SON*			NM_138927.3	
	11p15.2	*SOX6*	SNV	c.242C>G		Ser81∗
2	4q31.21	*SPRY1*	SV	c.16delC		Gln6fs*8
1	5q31.3	*SPRY4*	SNV	c.G478T		Glu160*
1	17q25.2	*TBCB*	Damaging missense (de novo)		NM_001281	Arg30Gly
1	15q21.3	*TCF12*	SNV	c.641C>G	NM_207036.1	Pro214Arg
n/a	15q21.3	*TCF12*	SNV	c.2024A>C	NM_207036.1	Glu675Ala
1	15q21.3	*TCF12*	SNV	c.971-8A>G	NM_207036.2	
1	5q14.1	*TENM2*	Damaging missense (de novo)		NM_001122679	Arg1813His
n/a	6p12.3	*TFAP2B*	Inherited SNV	c.G3A	NM_003221	Met1Ile
1	3p24.1	*TGFBR2*	SNV	c.1732T>A	NM_001024847	Ser578Thr
n/a	3p24.1	*TGFBR2*	SNV	c.1222C>A	NM_003242.5	Leu408Met
n/a	3p24.1	*TGFBR2*	SNV	c.569G>A	NM_003242.5	Arg190His
1	15q14	*THBS1*	Damaging missense (de novo)		NM_003246	Arg980Cys
1	9q21	*TMC1*	SV	Deletion	Chr9: 74.25–74.60 Mb	
n/a	12q24.11	*TRPV4*	SNV	c.496C>A	NM_021625.4	Leu166Met
1	11p14.1	*TTC12*	SNV	c.1816+1G>C	NM_017868	
1	11p11.2	*TWIST1*	Pathogenic SNV	c.439C>G	NM_000474	Gln147Glu
3	7p21.1	*TWIST1*	SNV	c.563C>T	NM_000474.2	Ser188Leu
1	7p21.1	*TWIST1*	SNV	c.170G>C	NM_000474.4	p.(Gly57Ala)
1	2q35	*Upstream of IHH*	SV	Duplications of 48 kb upstream of IHH	BER254251	
1	20p12.3	*downstream non coding region of BMP2*	SNV	7125642T>C	rs1884302	
2	1p13.2	*NOTCH2*	SNV	c.7223T>A	NM_024408	Leu2408His
n/a			SNV	c.929G>A		Gly310Asp
1	2q37		SV	Deletion		
1	16p13.11-p12.3		SV	16p13.11-p12.3 2.6Mbp microdeletion	hg19	
6			SV	7q11.23 deletion		
2	Xq12q21		SV	Xq13.1–q21.1 10Mbp duplication		
1			SV	add(4)(q32.3q33)		
1			SV	del(17)(q21.31)e		
1			SV	15q25.1–qter trisomy		

## Discussion

### Overview of the evidence

This systematic review synthesizes evidence from 97 studies investigating the relative contribution of genetic and environmental factors to the pathophysiology of sCS. Overall, the available literature is characterized by a predominance of descriptive and observational study designs, reflecting the rarity and heterogeneity of the condition. Genetic investigations were reported in the majority of studies, whereas environmental determinants were assessed less consistently and primarily within epidemiological frameworks.

### Genetic contribution to sagittal synostosis

The available evidence indicates that sCS has a markedly heterogeneous genetic basis and is unlikely to be explained by a single dominant molecular mechanism. Rather than being driven, in most cases, by recurrent high-penetrance mutations, sCS appears to arise from a broad spectrum of rare coding variants, structural rearrangements, regulatory alterations, and susceptibility alleles that converge on a limited number of developmental pathways controlling cranial suture patency.

This interpretation is consistent with the observation that most reported variants occur in single individuals or small cohorts, whereas only a few genes recur across independent studies.

Mutations in the classical CS genes are relatively uncommon in apparently isolated sCS. Variants in *TWIST1*,* FGFR1*, and *FGFR2* have been described, although each accounts for only a minor proportion of cases [62, 98, 120]. Likewise, rare variants in *ALX4* and *IGF1R* support a contribution of genes involved in osteoblast differentiation and suture growth [59, 62,119 Particularly, *ALX4* is involved in craniofacial development [62], anda variant in *ALX4* was found associated with a moderate form of frontonasal dysplasia type 1 with minute parietal foramina [126]. *ALX4* may be a potential target of *TWIST1* or even *TCF12*, both of which are required for calvarial development and suture closure [127, 128].

In contrast, synonymous or common variants of *FGFR3* have not shown clear pathogenic relevance, emphasizing the need to distinguish true disease-associated findings from background polymorphisms [23, 42]. Taken together, these studies suggest that the genetic architecture of sCS differs from that of the more recognizable monogenic CS syndromes.

The sCS may not always represent a purely isolated developmental anomaly but can also arise as part of broader syndromic or metabolic conditions. This is particularly evident in PHEX-related X-linked hypophosphatemic rickets (XLHR), in which sagittal suture involvement appears strikingly frequent and, in some cohorts, nearly constant [43, 81, 95]. These observations are relevant because they indicate that altered phosphate handling, bone metabolism, and cranial compliance may predispose specifically to premature sagittal suture closure.

More broadly, variants in genes such as *FGFR2*,* TCF12*,* ERF*,* KAT6B*,* ACTB*,* SH3PXD2B*,* SALL1*,* IFT140*,* SON*,* CASK*,* FOXP2*,* EXTL3*, and *SOX6* have often been identified in patients with additional dysmorphic, skeletal, neurodevelopmental, or systemic disorders [41, 47–49, 78, 84, 86, 90, 100, 101, 108–110]. This supports the view that at least a subset of cases initially classified as non-syndromic may in fact represent attenuated or atypical presentations within a wider syndromic continuum.

Despite this broad heterogeneity, the genes implicated in sCS do not appear random. Rather, they cluster in a restricted number of signalling pathways central to osteogenesis and cranial morphogenesis. Strong evidence supports a role for the BMP/SMAD pathway, particularly through *SMAD6*, whose variants represent one of the most consistent findings in midline NCS [62]; importantly, their penetrance appears to be modified by common regulatory variation near *BMP2*, supporting a two-locus model rather than a simple monogenic one [76]. Similarly, increasing evidence implicates WNT/β-catenin signalling through *AXIN1, AXIN2, and RUNX2*, suggesting that dysregulated osteoblast differentiation may be a recurrent biological mechanism in sCS [58, 105, 118, 121]. Additional convergence is seen in the Hedgehog pathway, involving *ALX4*,* IHH*, and *RAB23* [46, 72, 77], and in the RAS/MAPK cascade, given the association of sCS with RASopathies and variants in *BRAF*,* PTPN11*, and* MAP4K4* [61, 112, 122]. These data suggest that different genetic variations may converge on shared downstream mechanisms promoting premature osteogenic commitment of osteoblast precursors within the sagittal suture.

Additional studies also indicate that non-coding, regulatory, and structural genomic changes are relevant to disease susceptibility. Tandem duplications of upstream *IHH* enhancers, rare chromosomal microdeletions and microduplications, and CNVs involving regions that include genes such as *FGF2*,* SPRY1*,* SEMA5A*,* FBLN2*, and* TMEM43* support the concept that altered gene dosage and disrupted regulation can contribute to sCS even in the absence of pathogenic coding variants [64, 85, 87, 115, 124]. This is further reinforced by the identification of susceptibility loci near *BMP2* and within *BBS9*, as well as functional evidence implicating ciliogenesis and primary cilium biology in sagittal suture homeostasis [44, 76].

At the same time, Clarke et al. [25], Yoon et al. [123], and Sewda et al. [99] described numerous rare variants and variants of uncertain significance in genes including *FLNA*,* KMT2D*,* RECQL4*,* HUWE1*,* IDUA*,* IL11RA*,* MEGF8*,* FBN1*,* NOTCH1/2*,* ALPL*,* BMPER*, and* FREM1.* While individually difficult to interpret, these findings support an oligogenic or multifactorial model, in which several moderately penetrant variants may interact to increase susceptibility without being individually sufficient to cause disease.

The observation of discordant phenotypes in genetically identical twins carrying the same variant, as reported for *AXIN2* and *ERF*, further suggests that genetic predisposition alone may not fully determine the phenotype and that additional modifiers are likely involved [63, 118].

Overall, the evidence gathered confirmed that sCS exhibited a highly heterogeneous genetic structure, in which rare high impact variants coexisted with a multifactorial/oligogenic contribution.

### Environmental contribution to sagittal synostosis

#### Influence of biological sex on sCS insurgence

A well-known and consistent finding across the studies analyzed in this review is the marked male predominance (sum of all studies 5156 male and 1800 female) in sCS. The majority of the studies studied the odds ratio of male occurring CS with an OR ranging from 1.46 to 4.5 (with geometric mean of ~ 2.7) [2, 3, 15, 26, 50, 53, 74, 80, 82, 86, 96–98, 102, 103, 117]. These data have been found consistently during the years (from 1980 to 2024) and despite the population or region of birth. A possible explanation of this effect has been linked to possible sex-specific transcriptional factor that may alter osteoblasts differentiation signal mainly on males, while sCS occurrence in females have different genetic risk profiles [91].

### Conception, gestation, and birth as a risk factor for sCS

An important finding emerging from the studies reviewed here is that all stages of pregnancy are crucial for proper foetal development. Firstly, advanced maternal age (> 35 years) has been found to highly correlate with sagittal suture fusion, with an OR ranging from 2.01 to 2.32 [2, 82, 93, 102], while young maternal age has a protective effect OR 0.6 [56]. Only in one article advanced paternal age (> 40 years) has been found to pose as a risk factor for sCS, with an OR = 2.11 [102]. These effects can be explained by the increase of sperm and oocytes DNA damage and mutation with age, which then leads to birth defects. Interestingly, higher parental educational level (≥ 15 years of education) has been associated with an increased risk of sCS, with reported odds ratios ranging from 1.6 to 3.3 for maternal education [15] and up to 4.4 for paternal education [26]. This association may reflect differences in socioeconomic status, potentially leading to improved access to healthcare services and, consequently, higher rates of diagnosis in families with higher educational attainment compared to lower-income groups. Other possible risks during gestation that has been found in this systematic review are pre-pregnancy obesity (BMI ≥ 30) [93, 96], pre-term gestation (< 37 weeks) OR = 1.7–2.41 (*n* = 3) [29, 102]), number of previous births if two or more OR 1.29–1.39 (Lee 2012), if four or more the risks are been found to increase with a OR = 2.0 [15, 93], or twin gestation OR 2.1–3.89 [2, 29, 50]. At birth, there are some phenotypes that has been seen to correlate with sCS, from high weight at birth ≥ 4000 g (OR 1.61–2.91) [2, 82] to very low birth weight < 2500 g (OR = 1.5–2.32) [29, 102]), also to breech presentation (OR = 2.39) [63, 102]. These factors are suggested to be correlated with intra-uterine compression of the foetus, which ultimately can lead to wrong mechanical stimuli for the normal growth of the skull.

### Parental dietary intake and lifestyle risk factors in sCS

Maternal diet and lifestyle represent a critical point for correct foetal development and biology. Across epidemiological studies of NCS, factors such as maternal smoking, micronutrient intake, fertility treatment, and exposure to metals emerge as high risk factors, suggesting that alterations in the intrauterine metabolic and endocrine milieu may influence suture patency during critical windows of craniofacial development. Maternal smoke is one of the first and most documented risk factors for the insurgence of CS with OR 1.48–5.12 [15, 63, 69] for sagittal suture fusion. The effect can vary depending on both the timing of maternal smoking cessation (e.g. in the first, second, or third trimester) and the number of cigarettes smoked per day (with the highest risk observed in women smoking more than 15 cigarettes/day) [69].

An increased risk of sCS has been reported in pregnancies without a regular supplement intake, with an OR of 1.4. Conversely, intake of specific micronutrients has been associated with a reduced risk of sCS, including riboflavin (OR = 0.5), vitamin B6 (OR = 0.4), vitamin E (OR = 0.6), and vitamin C (OR = 0.7) [55]. It has been reported also that fertility treatments correlate to an increased presence of only sagittal synostosis if there is a twin gestation OR 1.6 [29].

Maternal thyroid disease has also been linked to an increased risk of CS, with reported ORs ranging from 1.3 to 2.11 [56, 93], consistent with the well-established role of thyroid hormones in skeletal development.

Another important factor that can influence skull growth and correct bone differentiation is maternal occupation, which can increase the exposure of metals [114, 118] and polycyclic aromatic hydrocarbons (PAHs) to the foetus [89]. The highest risk has been reported during the early months of pregnancy for PAH exposure (OR = 1.76). In contrast, exposure to metals such as tin, nickel, and chromium may represent a risk throughout pregnancy due to their effects on osteoblasts [114]. These exposures can alter *FGFR2* expression by decreasing methylation in the *FGFR2* regulatory region and enhancing access to the Sp1 transcription factor, leading to abnormal *FGFR2* upregulation and premature osteogenic differentiation [114].

Maternal alcohol consumption has been associated with odds ratios close to unity (OR ~ 0.8–1.0) for sCS [56, 65], but this does not imply a protective effect and does not modify existing recommendations to abstain from alcohol during pregnancy.

Ultimately, a limited number of studies have also investigated paternal lifestyle and occupational exposures. Occupations entailing higher potential contact with environmental agents (e.g. chemicals or ionizing radiation) have been hypothesized to exert direct mutagenic effects on sperm, potentially increasing the likelihood of germline mutations and, consequently, the risk of CS [52]. In this study, paternal employment as mechanics/repairmen was associated with increased odds across multiple suture types (OR range: 1.8–3.0), whereas no association was observed for sagittal synostosis among fathers working in agriculture/forestry (OR = 0.9) [52].

### Population and geographic contribution in sCS

Geographic location and place of birth have been associated with variation in CS risk, suggesting that contextual and environmental factors linked to where individuals are born, and parents reside may contribute to disease susceptibility. Collectively, most of the studies come from North America (34/97) and have 6501 (58.6%) of all sCS patients in this systematic review; secondly, there is Europe with (31/97 studies) and 1356 (12.2%) patients, then Asia and Oceania (12/97 & 7/97 studies) with 409/2426 (3.7% & 21.9%) sCS patients. An example of the link between area of living and CS has been shown by Schraw et al., where they found increased changes of patients to come from parents residing close to the US-Mexico border (aPR = 1.29) [96], and by Somolan et al., showing a correlation with patients living in more rural areas [103].

In addition, race and ethnicity have also been reported as modifiers of sCS risk. Several studies consistently report a higher prevalence among individuals of white race [29, 93], whereas lower risks have been observed in black (OR = 0.2), Hispanic (OR = 0.4), and other racial or ethnic groups (OR = 0.6) [56, 96]. Importantly, these associations are unlikely to reflect race-specific biological mechanisms per se but rather may act as proxies for underlying differences in environmental exposures, socio-economic conditions, access to prenatal care, nutritional factors, or gene-environment interactions that vary across populations. As such, race and ethnicity should be interpreted within a broader contextual framework rather than as independent causal risk factors.

### Limitations of evidence on environmental factors in sCS onset

Several limitations affect the assessment of environmental risk factors for sCS. Many studies, including case reports, case series, and small observational studies, are underpowered due to limited sample sizes, with only 29 out of 96 cohorts including more than 200 participants. This may lead to unstable effect estimates and reduced ability to detect true associations. Although useful for hypothesis generation, these study designs limit generalizability and causal inference [12, 25].

In addition, reliance on parental self-report introduces recall bias, particularly for early pregnancy exposures such as diet, supplement use, smoking, and environmental factors. Even among analytical studies, substantial heterogeneity in study design, exposure assessment, and outcome reporting complicates comparisons across cohorts [10].

Overall, these limitations underscore the need for larger, well-powered studies with prospectively collected exposure data to better clarify environmental contributions to sCS.

### Genetics-environment interplay

Rather than supporting a strict dichotomy between genetic and environmental aetiologies, current data indicate that sCS likely arises from a complex interplay between genetic susceptibility and environmental influences. The identification of rare and heterogeneous genetic variants, coupled with the absence of consistent high-penetrance mutations, implies that genetic predisposition alone is often insufficient to cause disease. Environmental factors may therefore act as modifiers or triggers in genetically susceptible individuals, influencing the timing or extent of sagittal suture fusion. This hypothesis is consistent with evidence of shared heritability across syndromic CS and NCS and with models of cranial development emphasizing the integration of molecular signalling and biomechanical forces. However, the scarce evidence of integrative studies simultaneously assessing genetic and environmental variables represents a major limitation in the current literature.

Twin studies provide some of the strongest evidence supporting gene-environment interplay in the development of sCS. Because monozygotic twins share (nearly) identical genetic backgrounds, reports of twin pairs in which both are affected by CS but with different sutures involved are particularly informative. These observations have been interpreted as consistent with a shared genetic susceptibility combined with non-shared environmental and/or stochastic factors, which may differentially perturb cranial signalling pathways and drive premature osteogenic differentiation in distinct sutures.

### Strengths and weaknesses

The main strengths of this study include its stringent and transparent methodology which increases reproducibility and the included sSC sample size of more than 11,086 patients. Moreover, restricting the review to a clearly defined CS subtype such as sCS increased the homogeneity of the included studies. The 26-year time frame covered by the selected studies provides a comprehensive overview of the existing evidence. The differences in study types, sequencing methodologies across the included records, resulted in challenging cross-study comparisons. Despite the variability in diagnostic approaches, which may have influenced the reported genetic diagnostic yield, a consistent pattern in the identified genes was observed across studies. Accordingly, the reported diagnostic yield should be interpreted in light of this methodological heterogeneity.

The included evidence derives from heterogeneous study designs and levels of evidence, including case reports, cohort studies, GWAS, and population-based studies. This heterogeneity is considered a limitation, and the findings should be interpreted cautiously because the available evidence is based on study designs with different risks of bias and different levels of methodological robustness.

Limiting the literature search to the PubMed database only may have resulted in a limited number of relevant publications being overlooked, although this is not expected to have had a major impact on the study overall.

## Conclusion

In conclusion, the evidence synthesized in this review supports a multifactorial model for sCS, in which neither genetic nor environmental factors appear to act as individually deterministic drivers in the etiopathogenesis of sCS, supporting a model in which multiple components likely interact. In recent years, there has been a growing trend toward the implementation of comprehensive genetic screening in patients with non-syndromic craniosynostosis, driven by advances in sequencing technologies and the increasing recognition of pathogenic variants in cases previously considered idiopathic. This expanding use of molecular diagnostics is progressively improving our understanding of the genetic architecture of sCS and may help clarify the extent to which genetic predisposition contributes to disease development, either independently or through interaction with environmental factors.

The marked male predominance, together with associations involving maternal age, pregnancy conditions, and modifiable exposures such as smoking, micronutrient intake, and environmental pollutants, highlights the importance of both biological and external determinants acting during critical windows of cranial development. Although genetic studies have identified numerous candidate genes and pathways involved in cranial morphogenesis, identifiable pathogenic variants account for only a small proportion of cases (~ 12.5% of approximately 2000 genetically sequenced sCS cases), while the vast majority (~ 96.3%) lack mutations in key signalling pathways, further supporting a prominent role for non-genetic contributions [3, 25, 42]. Despite this, the precise causal mechanisms remain incompletely understood. The predominance of negative or heterogeneous genetic findings underscores the complexity of the condition and suggests that genetic susceptibility alone is often insufficient to drive disease onset.

Overall, sCS appears to result from a complex interplay between rare genetic variants and environmental influences, rather than a single predominant etiological mechanism. However, current evidence is limited by heterogeneity across studies, potential biases in exposure assessment, and a lack of integrative analyses combining genetic and environmental data. Addressing current gaps through integrative, multidisciplinary research-combining genomic, epigenetic, environmental, and epidemiological data will be crucial to advancing the understanding of disease pathophysiology, improving risk stratification, and ultimately informing preventive and early intervention strategies.

## Supplementary information

Below is the link to the electronic supplementary material.

ESM 1  (PDF 272 KB)

ESM 2 (XLSX 238 KB)

## Data Availability

No datasets were generated or analysed during the current study.
